# Maternal Colonization Versus Nosocomial Transmission as the Source of Drug-Resistant Bloodstream Infection in an Indian Neonatal Intensive Care Unit: A Prospective Cohort Study

**DOI:** 10.1093/cid/ciad282

**Published:** 2023-07-05

**Authors:** Matthew L Robinson, Julia Johnson, Shilpa Naik, Sunil Patil, Rajesh Kulkarni, Aarti Kinikar, Vaishali Dohe, Swati Mudshingkar, Anju Kagal, Rachel M Smith, Matthew Westercamp, Bharat Randive, Abhay Kadam, Ahmed Babiker, Vandana Kulkarni, Rajesh Karyakarte, Vidya Mave, Amita Gupta, Aaron M Milstone, Yukari C Manabe

**Affiliations:** Division of Infectious Diseases, Department of Medicine, Johns Hopkins University School of Medicine, Baltimore, Maryland, USA; Division of Neonatology, Department of Pediatrics, Johns Hopkins University School of Medicine, Baltimore, Maryland, USA; Department of International Health, Johns Hopkins Bloomberg School of Public Health, Baltimore, Maryland, USA; Department of Obstetrics, Byramjee Jeejeebhoy Government Medical College, Pune, India; Department of Obstetrics, Byramjee Jeejeebhoy Government Medical College, Pune, India; Department of Pediatrics, Byramjee Jeejeebhoy Government Medical College, Pune, India; Department of Pediatrics, Byramjee Jeejeebhoy Government Medical College, Pune, India; Department of Microbiology, Byramjee Jeejeebhoy Government Medical College, Pune, India; Department of Microbiology, Byramjee Jeejeebhoy Government Medical College, Pune, India; Department of Microbiology, Byramjee Jeejeebhoy Government Medical College, Pune, India; Centers for Disease Control and Prevention, Atlanta, Georgia, USA; Centers for Disease Control and Prevention, Atlanta, Georgia, USA; Byramjee Jeejeebhoy Government Medical College, Johns Hopkins University Clinical Research Site, Pune, India; Byramjee Jeejeebhoy Government Medical College, Johns Hopkins University Clinical Research Site, Pune, India; Division of Infectious Diseases, Department of Medicine, Emory University School of Medicine, Atlanta, Georgia, USA; Byramjee Jeejeebhoy Government Medical College, Johns Hopkins University Clinical Research Site, Pune, India; Department of Microbiology, Byramjee Jeejeebhoy Government Medical College, Pune, India; Division of Infectious Diseases, Department of Medicine, Johns Hopkins University School of Medicine, Baltimore, Maryland, USA; Byramjee Jeejeebhoy Government Medical College, Johns Hopkins University Clinical Research Site, Pune, India; Division of Infectious Diseases, Department of Medicine, Johns Hopkins University School of Medicine, Baltimore, Maryland, USA; Division of Pediatric Infectious Diseases, Department of Pediatrics, Johns Hopkins University School of Medicine, Baltimore, Maryland, USA; Division of Infectious Diseases, Department of Medicine, Johns Hopkins University School of Medicine, Baltimore, Maryland, USA

**Keywords:** newborn, antimicrobial resistance, sepsis

## Abstract

**Background:**

Drug-resistant gram-negative (GN) pathogens are a common cause of neonatal sepsis in low- and middle-income countries. Identifying GN transmission patterns is vital to inform preventive efforts.

**Methods:**

We conducted a prospective cohort study, 12 October 2018 to 31 October 2019 to describe the association of maternal and environmental GN colonization with bloodstream infection (BSI) among neonates admitted to a neonatal intensive care unit (NICU) in Western India. We assessed rectal and vaginal colonization in pregnant women presenting for delivery and colonization in neonates and the environment using culture-based methods. We also collected data on BSI for all NICU patients, including neonates born to unenrolled mothers. Organism identification, antibiotic susceptibility testing, and next-generation sequencing (NGS) were performed to compare BSI and related colonization isolates.

**Results:**

Among 952 enrolled women who delivered, 257 neonates required NICU admission, and 24 (9.3%) developed BSI. Among mothers of neonates with GN BSI (n = 21), 10 (47.7%) had rectal, 5 (23.8%) had vaginal, and 10 (47.7%) had no colonization with resistant GN organisms. No maternal isolates matched the species and resistance pattern of associated neonatal BSI isolates. Thirty GN BSI were observed among neonates born to unenrolled mothers. Among 37 of 51 BSI with available NGS data, 21 (57%) showed a single nucleotide polymorphism distance of ≤5 to another BSI isolate.

**Conclusions:**

Prospective assessment of maternal GN colonization did not demonstrate linkage to neonatal BSI. Organism-relatedness among neonates with BSI suggests nosocomial spread, highlighting the importance of NICU infection prevention and control practices to reduce GN BSI.

## Teaser

Pre-delivery maternal sampling followed by neonatal observation did not identify a maternal source for neonatal gram-negative bloodstream infection. However, most neonatal gram-negative bloodstream isolates were closely related by sequencing to at least 1 other clinical isolate, raising concern for nosocomial transmission.

Worldwide, nearly half of early childhood deaths are in neonates, and up to one-half are due to infection [1]. More than 100 000 neonates die annually from infection in India, the highest absolute burden globally [2]. Neonatal sepsis is up to 10-fold more common in India than in high-income countries and has a mortality rate of up to 50% [3]. Gram-negative (GN) pathogens are the predominant cause of neonatal sepsis in India, where antimicrobial resistance (AMR) is highly prevalent [4, 5]. Neonatal sepsis caused by multi-drug resistant (MDR) organisms has limited treatment options and higher mortality, especially among neonates admitted to the neonatal intensive care unit (NICU) [5–8].

It is unclear whether neonatal infections with AMR GN pathogens are the result of early nosocomial transmission or reflect maternal colonization. Linkage between maternal colonization and subsequent neonatal colonization has been reported in high-income settings where AMR GN neonatal sepsis is uncommon [9–12]. In low- and middle-income country (LMIC) settings, where the prevalence of these infections is higher, some studies have investigated the linkage between maternal and neonatal colonization with AMR GN pathogens [13–15]. However, the role of maternal AMR GN colonization in neonatal sepsis risk has not been comprehensively assessed in India. We conducted a prospective cohort study to assess the association between maternal and environmental colonization and neonatal colonization and BSI with AMR GN pathogens in Western India. We hypothesized that pregnant women are colonized with AMR GN pathogens, which are transmitted to their neonates with resultant neonatal colonization and infection.

## METHODS

### Study Design and Population

We conducted a prospective cohort study from 12 October 2018, until 31 October 2019, to describe the role of maternal and environmental GN colonization in BSI among neonates admitted to the 60-bed NICU at Byramjee Jeejeebhoy Government Medical College (BJGMC) Sassoon General Hospital, a tertiary care facility in Pune, India. Infection control practices at BJGMC during the study period included oversight by an Infection Control Committee, which included microbiologists and infection preventionists, participation in a Comprehensive Unit-based Safety Program (CUSP) to improve hand hygiene, aseptic technique for invasive procedures, and medication and IV fluid preparation and administration [16], and facility-level antimicrobial stewardship without capacity for prospective audit and feedback. Women admitted to Labor & Delivery with risk factors for delivering children developing neonatal sepsis who provided consent were enrolled. Neonates were followed until discharge, transfer, or death. Inclusion criteria for enrollment were expected preterm delivery (<37 weeks gestation), low birth weight (based on estimated fetal weight), prolonged rupture of membranes (>18 hours prior to delivery), meconium-stained amniotic fluid, history of febrile illness within 2 weeks prior to presentation, or otherwise deemed to be high-risk for neonatal sepsis by clinicians. Neonates from enrolled mothers who were admitted to the NICU were included in Cohort A (Supplementary Figure 1).

A separate cohort study prospectively observed all neonates admitted to the BJGMC NICU to characterize the epidemiology of BSI [5]. Neonates admitted to the BJGMC NICU between 12 October 2018, and 31 October 2019 but not enrolled into Cohort A were included in Cohort B to compare BSI strain relatedness among all neonates admitted to the NICU during the study period (Supplementary Figures 1 and 2). This study was approved by the BJGMC Ethics Committee, the Indian Council of Medical Research, and the Johns Hopkins Medicine Institutional Review Board.

### Assessment for Neonatal Bloodstream Infections

Blood cultures were obtained at the discretion of treating clinicians and processed per routine clinical care. The BJGMC Microbiology Laboratory performs routine microbiology tests, including organism identification and antimicrobial susceptibility testing (AST), and is accredited by the Indian National Accreditation Board for Testing & Calibration Laboratories. BSI was defined as positive blood culture with a known neonatal pathogen and classified as early onset if collection of positive blood culture specimen was on day of life (DOL) 0–2 and late onset if specimen collection was on DOL 3 or later. Isolates were considered “drug-resistant” if they were non-susceptible to third or fourth generation cephalosporins or piperacillin-tazobactam.

### Assessment for Maternal, Neonatal, and Environmental Colonization

Colonization with GN organisms resistant to carbapenems or 3rd generation cephalosporins was assessed in maternal and neonatal samples. Maternal vaginal and rectal swabs were collected at time of enrollment and repeated at delivery if >6 hours after enrollment. Staff collected skin and peri-rectal swabs from neonates admitted to the NICU on DOL 0, 3, 7, and weekly until NICU exit. Environmental colonization was assessed with weekly sampling of unit sinks and the immediate neonatal care environment. Samples were collected using the Eswab collection system (COPAN FLOQSwabs, 1 mL Liquid Amies medium), which were aliquoted and frozen at −80°C (Supplementary Methods). For neonates who had BSI, maternal, neonatal, and environmental samples obtained up to the week of BSI onset were thawed. Colonization sample aliquots were evaluated in duplicate with and without enrichment in tryptic soy broth for 4 hours.

Samples were plated onto MacConkey agar plates with meropenem, ceftriaxone, and ceftazidime discs. Representative colonies growing near antibiotic discs were analyzed using VITEK for organism identification and antimicrobial susceptibility testing. Isolates were stored at −80°C pending next-generation sequencing (NGS).

### Next-generation Sequencing and its Interpretation

GN isolates from clinical BSI samples and those recovered from associated maternal colonization and environmental swabs were selected for sequencing. DNA was extracted from bacterial isolates using the QIAamp DNA Mini Kit. DNA extraction quality was assessed using Qubit and QIAXpert. NGS was performed using Illumina HiSeq with a read length of 2 × 150 bp and target sequencing depth of 100×. After quality control, de novo gene assembly was performed (Supplementary Methods). AMR genes were identified using the ResFinder [17], PointFinder [18], Comprehensive Antibiotic Resistance Database (CARD) [19,] and ARG-ANNOT [20] databases. Core genomes among isolates of the same species were assembled to visualize phylogenetic relationships with maximum likelihood trees [21]. Species represented by fewer than 4 isolates were characterized by hierarchical clustering of pairwise single nucleotide polymorphism (SNP) distance. Evaluations of strain relatedness may produce inconsistent results dependent on the subjective choice of reference genome or parameters for core genome construction [22]. Strain relatedness was therefore evaluated by quantifying pairwise SNPs between each pair of isolates of the same species without requirement for reference genome selection or core genome creation [23] and displayed as distance matrices and force-directed network graphs.

## RESULTS

The study recruited 1051 women admitted to Labor & Delivery with neonatal sepsis risk factors (Supplementary Figure 1) into Cohort A; 952 proceeded to deliver. Median gestational age at the time of delivery was 36.1 weeks (interquartile range [IQR] 34.6–38.9; Table 1). There were 13 (1.3%) stillbirths and 983 (98.7%) liveborn neonates, of whom 257 (26%) required NICU admission (Supplementary Figure 1 and Supplementary Table 1). Antepartum antibiotics were administered to 737 (79%) of women with live births. The most common antibiotics administered were metronidazole, cefotaxime, and ampicillin for both cesarean and vaginal delivery. During the recruitment period for Cohort A, there were 1301 additional neonates admitted to the NICU who were not born to mothers who had been prospectively enrolled into Cohort A (Supplementary Figure 2).

**Table 1.** Clinical Characteristics Among Neonates Admitted to the NICU by Cohort Type, Pune, India, October 2018 to October 2019

	All Neonates n = 1558	Cohort A n = 257	Cohort B n = 1301
Maternal age in years, median (IQR)	23.0 (21.0–26.0)^a^	23.0 (21.0–26.0)	23.0 (21.0–26.0)^a^
Maternal antepartum antibiotic use, n (%)	724 (86.8%)^a^	215 (83.7%)	509 (88.2%)^a^
Male, n (%)	891 (57.2)	142 (55.3)	749 (57.6)
Gestational age in weeks, n (%)			
<28	34 (2.2)	6 (2.3)	28 (2.2)
28–32	321 (20.6)	100 (38.9)	221 (17.0)
33–36	400 (25.7)	110 (42.8)	290 (22.3)
≥37	562 (36.1)	41 (16.0)	521 (40.0)
Unknown	241 (15.5)	0 (0.0)	241 (18.5)
Birth weight in grams, n (%)			
<1000	57 (3.7)	6 (2.3)	51 (3.9)
1000–1499	236 (15.1)	57 (22.2)	179 (13.8)
1500–2499	706 (45.3)	149 (58.0)	557 (42.8)
>2500	504 (32.3)	45 (17.5)	459 (35.3)
Unknown	55 (3.5)	0 (0.0)	55 (4.2)
Multiple gestation, n (%)	115 (7.4)	48 (18.7)	67 (5.1)
C-section, n (%)	383 (32.9)^a^	78 (30.4)	305 (33.7)^a^
PPV at delivery, n (%)	223 (14.3)	43 (16.7)	180 (13.8)
Day of life at NICU admission	0 (0.0–2.0)	0.0 (0.0–0.0)	0.0 (0.0–3.0)
Mechanical ventilation on admission, n (%)	127 (8.2)	17 (6.6)	110 (8.5)
Central line on admission, n (%)	57 (3.7)	6 (2.3)	51 (3.9)
Pressors on admission, n (%)	1112 (71.4)	212 (82.5)	900 (69.2)
Antibiotics on admission, n (%)	493 (31.6)	118 (45.9)	375 (28.8)

Characteristics of neonates on admission to the NICU by cohort. Cohort A includes neonates born to mothers prospectively enrolled prior to delivery; Cohort B includes neonates admitted to the NICU during the study period but not enrolled in the maternal colonization study and observed for bloodstream infection (BSI) as part of a parent cohort study. Inclusion criteria for Cohort A were crafted to preferentially enroll mothers with increased risk of giving birth to a neonate that would require intensive care. Abbreviations: IQR, interquartile range; NICU, neonatal intensive care unit; PPV, positive pressure ventilation.

Cohort B data are unavailable for maternal age in 500 patients, mode of delivery in 395 patients, and maternal antepartum antibiotic use for 724 patients.

### Neonatal Characteristics

Among 257 neonates admitted to the NICU in Cohort A, median gestational age was 33.9 weeks (IQR 31.9–36.0), and median birth weight was 1700 grams (IQR 1500–2200). Seventy-eight (30.4%) were delivered via cesarean delivery (Table 1). On admission, 118 (45.9%) received antibiotics. Clinicians obtained at least 1 blood culture for 115 (44.7%) neonates. Twenty-four neonates (9.3% of Cohort A, 20.9% of those with blood cultures) developed BSI; 21 (87.5%) had GN BSI of which 15 (71.4%) were resistant to 3rd/4th generation cephalosporins or piperacillin/tazobactam and 8 (38.1%) were non-susceptible to carbapenems (Supplementary Table 2). Age at BSI onset ranged from 0 to 30 days of life; 5 (23.8%) were early onset, and 16 (76.2%) were late onset BSI.

The median gestational age for the 1301 neonates in Cohort B was 36.9 (IQR 33.1–38.6) weeks and median birth weight was 2200 (IQR 1600–2700) grams. Among the 906 neonates for whom data were available, 305 (33.7%) were delivered by C-section. Thirty neonates (2.3%) developed 36 GN BSI of which 27 (75%) were resistant to 3rd/4th generation cephalosporins or piperacillin/tazobactam and 13 (36.1%) were non-susceptible to carbapenems.

### Maternal Colonization, Neonatal Colonization, and Environmental Sampling

**Figure 1.**
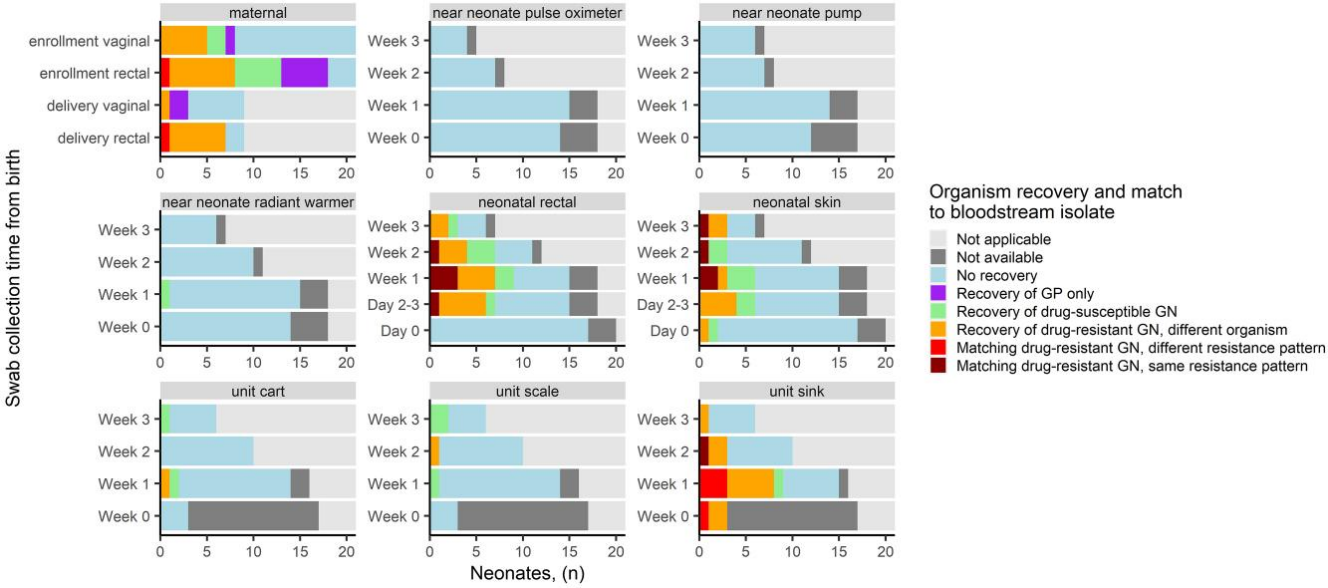
Organism recovery from swabs and match to 21 neonatal BSI isolates from Cohort A by sample source and time of collection from birth, Pune, India, October 2018 to October 2019. Each tile represents a single time point and swab sample source for an individual neonate. There were no exact matches between neonatal BSI isolate species and drug susceptibility profile to maternal colonization swabs. Drug-resistant GN defined as those resistant to 3rd/4th generation cephalosporins or piperacillin/tazobactam. Abbreviations: BSI, bloodstream infection; GN, gram-negative.

Among the 21 maternal samples associated with a neonatal GN BSI, there was growth of at least one species of drug-resistant GN organisms in rectal samples for 10 (47.7%) mothers and in vaginal samples for 5 (23.8%) mothers (Figure 1). Among 10 mothers with drug-resistant GN rectal colonization, there was a species match to the associated neonatal BSI for two mothers, though the pattern of drug resistance determined by susceptibility to 3rd generation cephalosporins and carbapenems was different; the remaining 8 were a different species. No drug-resistant GN organisms recovered from maternal vaginal specimens matched the species of associated neonatal BSI for those evaluated in Cohort A.

### Strain Relatedness in Clinical BSI Samples by Next-generation Sequencing

**Figure 2.**
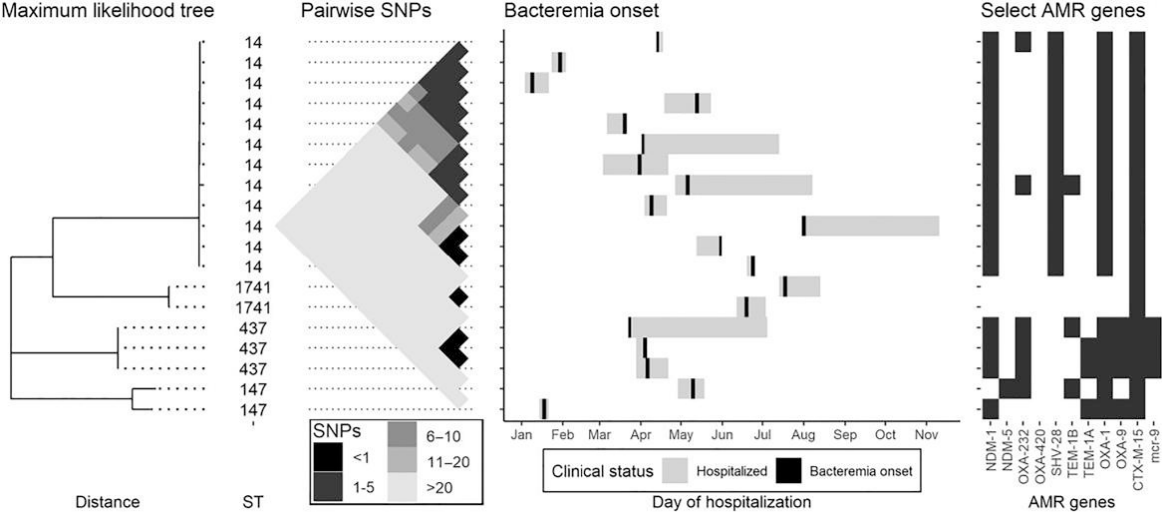
Strain relatedness, dates of hospitalization, bacteremia onset, and select AMR genes for 19 neonates (Cohort A, n = 6; Cohort B, n = 13) with *Klebsiella pneumoniae* bloodstream infection during the study period, Pune, India, October 2018 to October 2019. Strain relatedness is expressed on the left as a rooted maximum likelihood phylogenetic tree followed by the ST assigned by MLST. Next to the right, pairwise SNPs between each pair of isolates is presented as a rotated distance matrix with the diagonal aligned with the corresponding isolate from the phylogenetic tree. Corresponding period of hospitalization and bacteremia onset are displayed on the right followed by presence of select AMR genes. Abbreviations: AMR, antimicrobial resistance; MLST, multilocus sequence typing; SNP, single nucleotide polymorphism; ST, sequence type.

**Figure 3.**
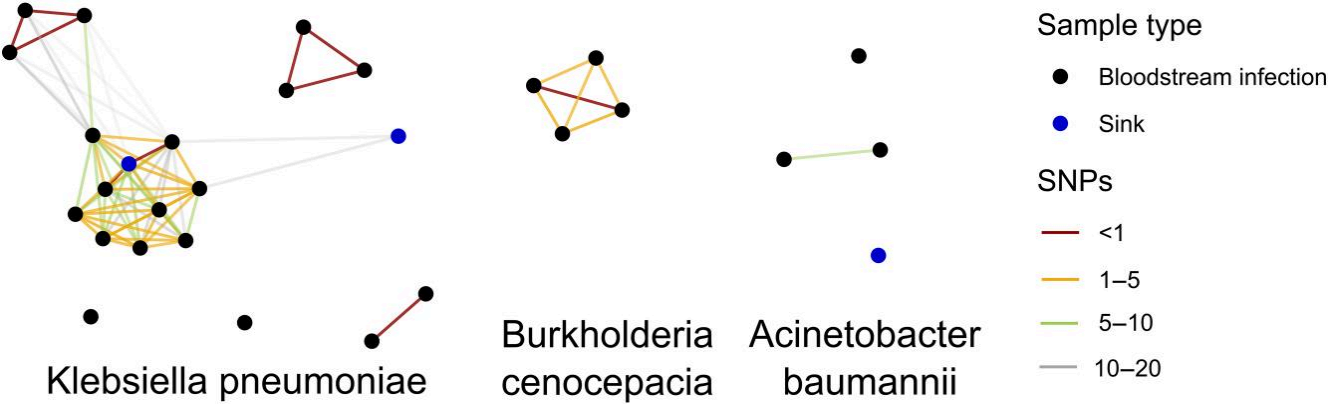
Network graph of pairwise SNPs between *Klebsiella pneumoniae*, *Burkholderia cenocepacia,* and *Acinetobacter baumannii* isolates from Cohort A and B neonatal bloodstream infections and sink samples with available sequencing data, Pune, India, October 2018 to October 2019. Relative position of unconnected isolates is arbitrary. An additional 10 BSI isolates of other species without linkage to other isolates are not plotted. Abbreviations: BSI, bloodstream infection; SNP, single nucleotide polymorphism.

Among 54 distinct clinical GN isolates obtained from 51 neonates in Cohorts A and B, 42 isolates were available for sequencing, organisms were unrecoverable from 2 stored specimens, and sequencing quality control failed for 3. NGS results were available for 37 BSI isolates including 16 from Cohort A and 21 from Cohort B. Among 19 *Klebsiella pneumoniae* BSI isolates with available NGS results, 4 sequence types were observed (14, 437, 1741, and 147) (Figure 2). There were 12 *K. pneumoniae* ST 14 isolates, which all had an SNP distance of <5 to the nearest neighbor (Figures 2 and 3). Three *K. pneumoniae* isolates were ST 437, which all had an SNP distance of ≤1 to the nearest neighbor. Two *K. pneumoniae* ST 1741 isolates had an SNP distance of 0. Two *K. pneumoniae* ST 147 isolates were separated by an SNP distance of 3438. Clusters of closely related *K. pneumoniae* BSI isolates were obtained from neonates who were hospitalized within 1 month of another neonate in the same cluster (Figure 2). All 4 *Escherichia coli* isolates were of different sequence types (361, 1193, 38, and 73; Supplementary Figure 3A). There were four isolates identified as *Burkholderia cenocepacia*; all identified as ST 824 (Supplementary Figure 3B) and had SNP distances of ≤4 to the nearest neighbor (Figure 3). Two of the 3 *Acinetobacter baumannii* isolates were ST 25 and had a SNP distance to each other of 8; the other was ST 85 (Figure 3 and Supplementary Figure 3C). An additional *Acinetobacter* isolate was identified as *A. indicus*. Both *Enterobacter cloacae complex* isolates were identified as *Enterobacter hormaechei* by NGS (ST 93 and ST 346). The 2 *Pseudomonas aeruginosa* isolates were of different ST (1684 and 2615). An additional isolate was identified as *Pseudomonas stutzeri*. In summary, 21 (57%) of the 37 BSI isolates with available NGS data showed an SNP distance of ≤5 to another clinical BSI isolate (Figure 3).

### Strain Relatedness Including Maternal and Environmental Samples

Two maternal rectal *K. pneumoniae* isolates resistant to ceftriaxone or meropenem and underwent NGS were found to have sequence types 551 and 34 and did not match any sequenced neonatal BSI isolates. There were 2 *K. pneumoniae* ST 14 isolates recovered from sink traps, the most common ST encountered in BSI isolates; their nearest neighbor among ST 14 BSI isolates by pairwise SNP distance were 1 and 15 (Figure 3).

### Identification of AMR Genes and Plasmids

Among BSI isolates, NDM-1 was found in 16 *K. pneumoniae* isolates, 1 *A. baumannii* isolate, and 1 *E. hormaechi* isolates (Figure 2, Supplementary Figure 3). NDM-5 was identified in 1 *K. pneumoniae* BSI isolate. NDM-1 was found in all sink isolates with available NGS data—2 *K. pneumoniae* and 1 *A. baumannii* isolates. No NDM were found in any sequenced maternal samples. OXA-232 was found in 6 *K. pneumoniae* BSI isolates and 1 *K. pneumoniae* sink isolate; OXA-420 was found in 1 *A. baumannii* BSI isolate. The mcr-9 gene was detected in 3 *K. pneumoniae* BSI isolates and 1 *A. baumannii* sink isolate. The most detected plasmid groups in Enterobacterales were IncFII and IncFIB (Supplementary Table 3).

## DISCUSSION

Prospective evaluation of maternal colonization prior to delivery and observation for subsequent neonatal BSI did not find evidence of maternal colonization as the source for AMR GN organisms in a cohort of Indian neonates. Most neonatal BSI isolates, however, showed a high degree of strain relatedness to other neonatal BSI occurring during a similar timeframe, suggesting that nosocomial transmission fuels AMR in this setting. A high degree of strain relatedness among neonatal BSI isolates was also found in secondary analysis of a large multinational cohort of neonatal BSI in LMICs [24]. These findings suggest that nosocomial rather than vertical transmission may be the primary driver of drug-resistant GN BSI in this setting.

Among 21 neonates with GN BSI, no isolates matched the species and resistance profile of bacteria isolated in maternal samples, suggesting that maternal colonization is unlikely to be the primary source of AMR in neonatal GN BSI in our study. Prior efforts have not consistently identified mother-child transmission of drug-resistant GN colonization in LMICs. A meta-analysis reported a MDR GN transmission rate of 27% from colonized mothers to neonates [25], but only 2 of 8 studies were conducted in LMICs and none in India. A study of very low birth weight (VLBW) inborn neonates in India showed that by the first week of life 68% were colonized with extended-spectrum-beta-lactamase (ESBL)-producing GN bacteria and 5% with carbapenem-resistant organisms (CRO), but no association between maternal and neonatal colonization was identified [15]. Studies in Sri Lanka have shown concordant *Enterobacteriaceae* colonization in <10% mother-neonate pairs and that most neonatal isolates are not clonally related to maternal isolates [13, 14]. The Burden of Antibiotic Resistance in Neonates from Developing Societies (BARNARDS) study recently reported eight clonal carbapenem-resistant isolate pairs in maternal and neonate gut microbiota samples, suggesting that mother to neonate transmission of colonizing resistant organisms does occur infrequently, although the study did not link colonization directly to BSI [26].

Lack of evidence of mother-to-child transmission of GN organisms causing BSI suggests that neonates must acquire these organisms during hospitalization. We found that among 37 GN BSI cases with available sequencing data, more than half showed fewer than 5 SNPs to another BSI case, suggesting likely association with at least 1 other case of GN BSI during the study period. Genetic similarity between isolates from neonates with GN BSI supports nosocomial transmission as the primary driver of GN BSI. Nearly all *K. pneumoniae* BSI isolates showed genetic similarity to isolates obtained from other neonates with *K. pneumoniae* BSI hospitalized within the same month. *K. pneumoniae* is an organism of global concern and has been linked to outbreaks in LMIC NICUs [27–29]. The National Institute for Health Research Global Health Research Unit (GHRU) on Genomic Surveillance of AMR has prioritized sequencing of *K. pneumoniae* isolates in consortium countries, including in India. The *K. pneumoniae* ST identified in this study were among the most common ST found at the GHRU surveillance site in another part of India [30].

Although other species of GN causing BSI had fewer isolates to compare in our cohort, clusters of closely related *B. cenocepacia* and *A. baumannii* were identified, which in aggregate with *K. pneumoniae* isolates constituted the majority of observed and analyzed BSI. Although worldwide surveys of GN bacterial strain types do show regional and national clustering of strain types, the SNP distance of clusters of GN BSI in this study show closer relatedness than expected by regional clustering [24, 31]. Close genetic similarity between isolates presented here demonstrates that nosocomial transmission is common in this setting. The BARNARDS study also reported related clusters of neonatal gut colonization [26] and BSI, suggesting that nosocomial transmission is common in other LMIC facilities. Study of the maternal and neonate gut microbiota in the BARNARDS study revealed clusters of clonal carbapenem-resistant isolates among neonatal samples and some maternal-neonate pairs [24].

Given our cohort's predominance of late-onset BSI and clustering of neonatal BSI isolates, AMR GN pathogen transmission likely occurs primarily within the NICU. Prevention of NICU healthcare-associated infection (HAI) relies on strong infection prevention and control (IPC) policies and practices [32]. In the NICU, special considerations for IPC exist, due to a particularly vulnerable population, as well as specialized equipment and practices unique to neonates [33]. In LMIC settings, additional challenges include unreliable water supply, reuse of single-use equipment, insufficient personal protective equipment and capacity for transmission-based isolation, high patient-to-staff ratio, and overcrowding of healthcare facilities [33, 34]. Strategies recommended by the World Health Organization to reduce risk of nosocomial transmission of AMR GN pathogens include implementation of multimodal IPC strategies, including hand hygiene, AMR surveillance, use of contact precautions and patient isolation, and environmental cleaning, although capacity is resource-dependent [35–38].

Efforts to reduce neonatal sepsis mortality in LMICs have been constrained by the fundamental knowledge gap of whether maternal colonization or nosocomial spread is responsible for the high burden of AMR in GN BSI. The concern that maternal colonization serves as the reservoir for AMR in subsequent neonatal GN BSI is driving investment in preventative efforts including investigation of maternal vaccination to reduce colonization with AMR organisms such as *K. pneumoniae* [39]. The findings from this study suggest that interventions to reduce maternal AMR colonization may have limited impact in settings where maternal rectal or vaginal colonization is not the chief driver of these infections.

Performance at a single site may limit our study's generalizability. Further study is needed in larger populations to determine the relationship between maternal colonization and neonatal sepsis, including in non-hospital settings. The frequency of neonatal sampling could have missed mother-to-neonate colonization transmission. The small number of neonatal GN BSI cases were insufficient to address other factors such as antibiotic use which may impact the risk of a neonate developing an AMR infection. Limited bacterial recovery in environmental samples other than sink traps fails to reveal the environment as a transmission reservoir. The study includes relatively few BSI cases compared to adult studies, but unique risk factors and infection control challenges for BSI prevention in neonates justifies focused study of this vulnerable population. Study strengths include prospective enrollment of mothers prior to delivery and evaluation of strain relatedness among multiple species and sequence types. Most reports of strain relatedness occur in the context of outbreak investigation and do not characterize strain relatedness in the broader context of BSI.

To reduce the global burden of neonatal sepsis and associated mortality due to AMR GN pathogens, elements of healthcare delivery responsible for nosocomial transmission must be identified to guide prevention. Our study did not show evidence of mother-to-child transmission of drug-resistant GN organisms causing BSI but did show that nosocomial transmission is a significant factor in transmission of these organisms, including *K. pneumoniae*, a major cause of neonatal sepsis in India and other LMIC settings. IPC initiatives must be prioritized to reduce HAI among hospitalized neonates in LMIC settings.

## Supplementary Data

Supplementary materials are available at *Clinical Infectious Diseases* online. Consisting of data provided by the authors to benefit the reader, the posted materials are not copyedited and are the sole responsibility of the authors, so questions or comments should be addressed to the corresponding author.

## Supplementary Material

ciad282_Supplementary_Data

## Notes

***Acknowledgments.*** The authors wish to thank the staff of the BJGMC Labor & Delivery ward and NICU for their support of this study.

***Supplement sponsorship.*** This article appears as part of the supplement “The Evolving Challenges of Antibiotic Resistance in Low- and Middle-Income Countries: Priorities and Solutions,” sponsored by the U.S. Centers for Disease Control and Prevention, and Health Security Partners.

***Financial support.*** This work was supported by the US Centers for Disease Control and Prevention (Safe Healthcare, Epidemiology, and Prevention Research Development Program Domain 7, contract number 200-2016-91781) and the National Institutes of Health (grant number UM1AI104681 to M. L. R., grant number K23HD100594 to J. J., grant numbers UM1AI069465-13 and 1R01AI43748-01A1 to V. M., grant numbers UM1AI069465, UM1AI068632, and UM1AI068636 to A. G., and grant number K24AI141580 to A. M. M.).
