# Supplementary material for: Maternal Colonization Versus Nosocomial Transmission as the Source of Drug-Resistant Bloodstream Infection in an Indian Neonatal Intensive Care Unit: A Prospective Cohort Study
Source: Clin Infect Dis. 2023 Jul 5;77(Suppl 1):S38–45. doi: 10.1093/cid/ciad282 (PMC10321698; doi:10.1093/cid/ciad282)

**SUPPLEMENTARY MATERIALS**

**Supplementary Methods**

**Environmental sample collection protocol**

Samples were collected from neonatal surrounds upon neonate admission to the NICU, weekly after admission until discharge using Copan ESwabs. Swabs of sink traps, radiant warmers, IV/feeding pumps, and pulse oximetry monitors, as well as negative control swabs, were collected weekly. Prior to sampling of equipment, ESwabs were moistened with neutralizing buffer to counteract the residual bactericidal effect of cleaning compounds on equipment. Each target was sampled with one ESwab in multiple sweeps.

Components of shared equipment were sampled in a standardized fashion according to a written standard operating procedure. For the radiant warmer, staff were instructed to swab the two buttons on the back of the radiant warmer and the guardrails. For the IV/feeding pump, staff sampled the screw cap, the three buttons, and the syringe plunger. For the pulse oximetry and respiratory support monitor, staff sampled the control knob as well as the alarm indicators. When positive pressure ventilatory support devices were present, the same swab was used to also sample the control knob. Sink traps were sampled by inserting the tip of the swab as far into the sink drain as possible and taking one sweep around the rim of the pipe after removing the sink drain cover. The equipment trolleys were swabbed by first sampling one circle around the top of each of the 4 posts and one line down each of the three connecting top rods.

**Genome assembly and strain relatedness**

Quality control of NGS reads was performed using FastQC (<http://www.bioinformatics.babraham.ac.uk/projects/fastqc>). Taxonomic characterization and distribution of NGS reads was performed using Kraken2 and Bracken.[16, 17] Aggregate NGS quality was reviewed using MultiQC.[18] Multilocus sequence typing (MLST) according to the PubMLST database (https://pubmlst.org/) [19] was performed using mlst (https://github.com/tseemann/mlst). De novo gene assembly was performed using Unicycler (v0.4.8)[20] which performs read correction, SPAdes[21] optimization, Pilon polishing,[22] multiplicity identification, and bridge construction. Bacterial genomes were annotated with Prokka (v1.13.4).[23] AMR genes were identified among the ResFinder[24], PointFinder[25], Comprehensive Antibiotic Resistance Database (CARD)[26] and ARG-ANNOT [27] databases using GAMMA (v2.1).[28] Core genomes among isolates of the same species were assembled with Roary (v3.13.0) to visualize phylogenetic relationships.[29] Maximum likelihood phylogenetic trees were constructed using RAxML-NG (v0.8.0).[30] Species represented by fewer than 4 isolates were characterized by a hierarchical clustering of pairwise single nucleotide polymorphism (SNP) distance. Rooted phylogenetic trees were created using ggtree (v3.4.0)[31]

Evaluations of strain relatedness may produce inconsistent results dependent on the subjective choice of reference genome or parameters for core genome construction.[32] Strain relatedness was therefore evaluated by quantifying pairwise SNPs between each pair of isolates of the same species without requirement for reference genome selection or core genome creation.[33] Pairwise SNPs were quantified by aligning reads of one isolate to the de novo assemblies of the other isolate of the pair using snippy (https://github.com/tseemann/snippy), removing self-SNPs, reversing the roles of the isolates, and reporting the median of bidirectional comparison.[33] Pairwise SNP distances were displayed as distance matrices and force-directed network graphs using ggraph [34] and igraph [35].

**Supplementary Figures**

**
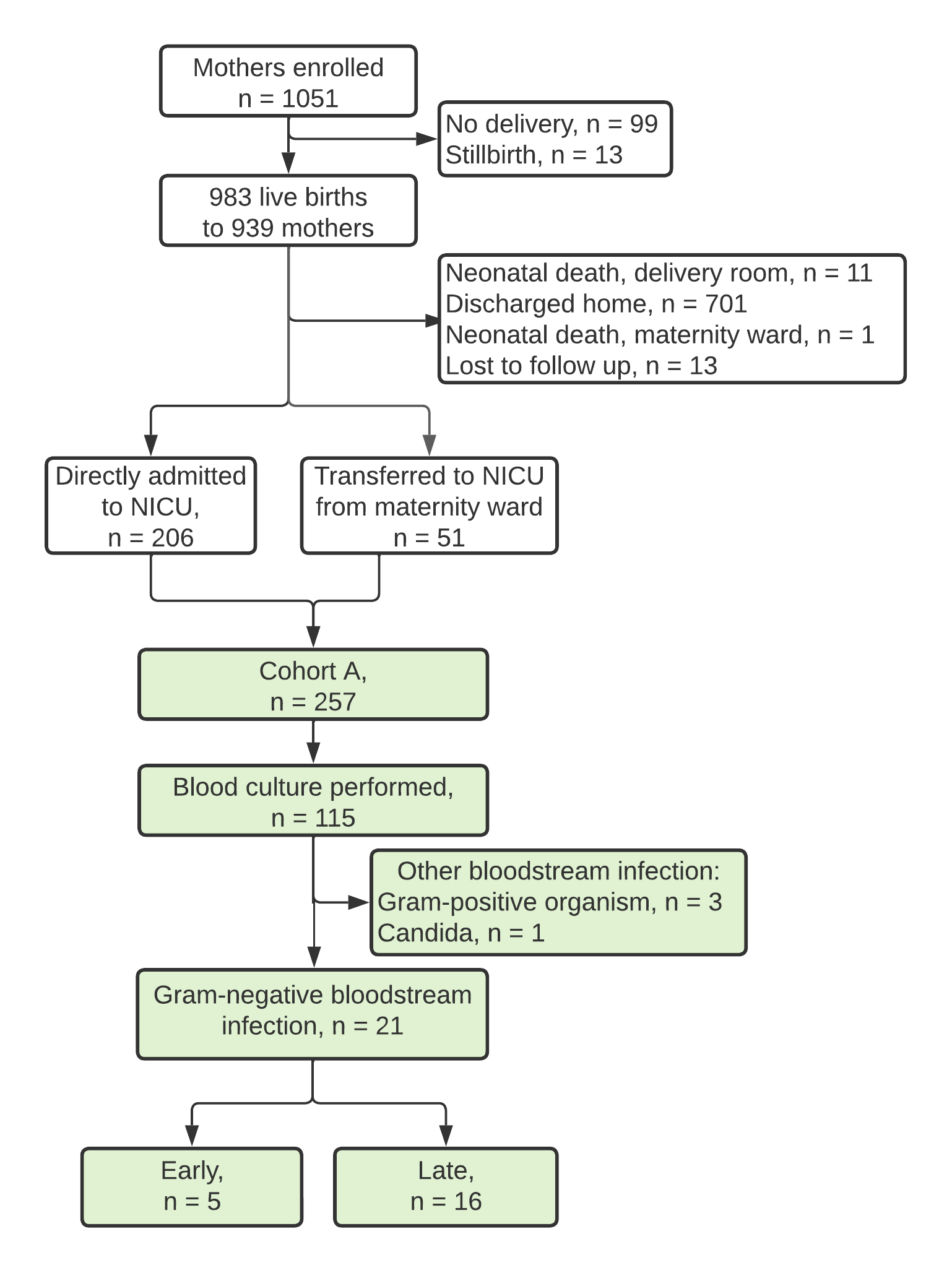
**

**Supplementary Figure 1. Study flow diagram, Cohort A, Pune, India, October 2018-October 2019.** Early onset bloodstream infections were defined as positive blood culture on specimen collected on DOL 0-2; late onset bloodstream infections were defined as positive blood culture on specimen collected on DOL 3 or later. Enrollment occurred October 12, 2018, to October 31, 2019. Abbreviations: NICU – neonatal intensive care unit.


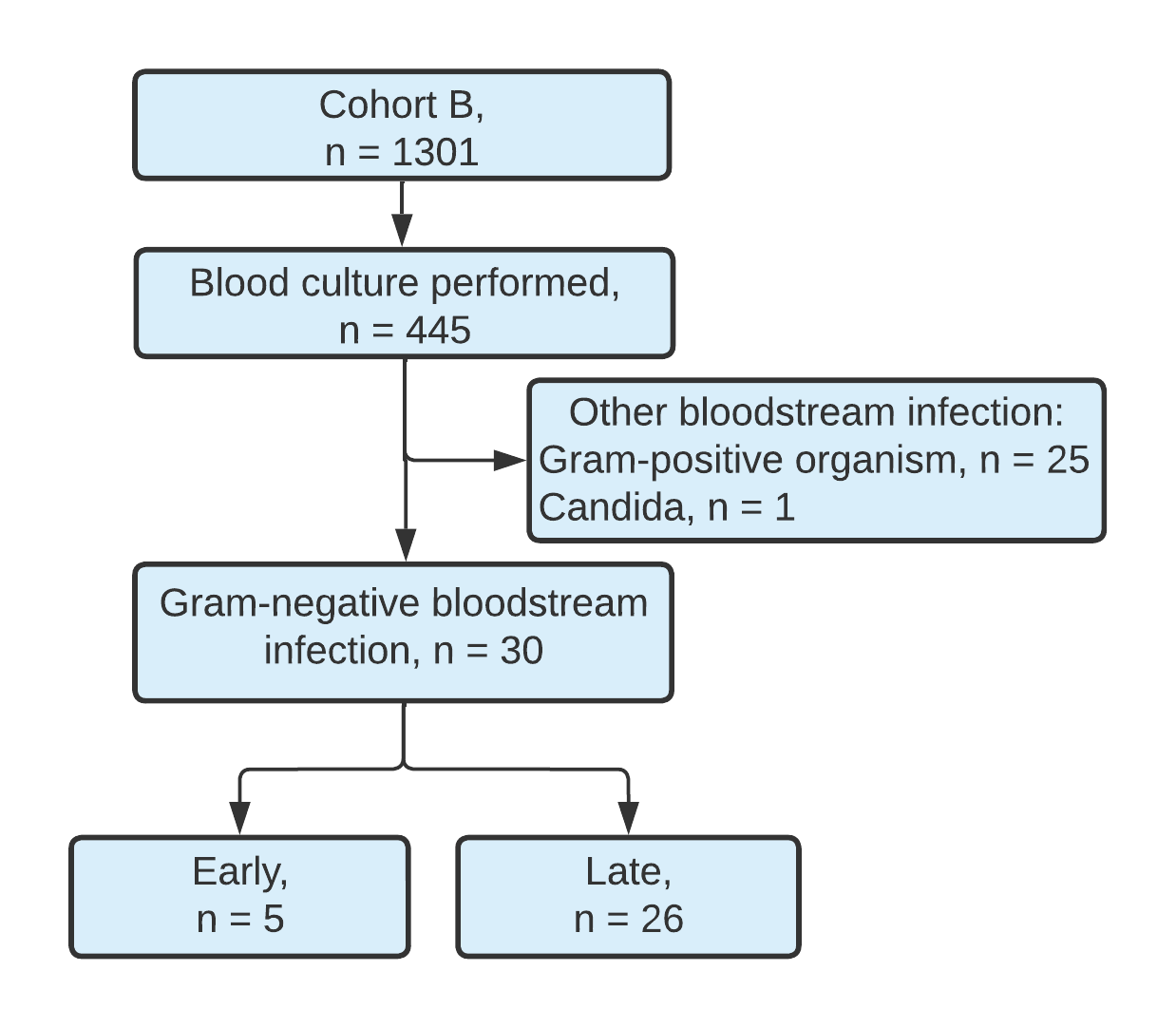


**Supplementary Figure 2. Study flow diagram, Cohort B,** **Pune, India, October 2018-October 2019.** Early onset bloodstream infections were defined as those occurring on day of life 0-2; late-onset bloodstream infections were defined as those occurring on day of life 3 or later. One patient had both early and late onset infections. Prospective observation occurred for neonates admitted to intensive care October 12, 2018, to October 31, 2019.

**Supplementary Table 1**. **Maternal characteristics by neonatal disposition for 939 women, Cohort A, Pune, India, October 2018-October 2019**

|  | **All women with live births**  **(n=939)** | **Women with neonatal death in DR**  **(n=10)** | | **Women with neonate admitted to NICU**  **(n=237)** | **Women with neonate not admitted to NICU**  **(n=692)** |
| --- | --- | --- | --- | --- | --- |
| Maternal age in years, median (IQR) | 23.0 (21.0-26.0) | 22.5 (22.0-24.5) | | 23.0 (20.0-26.0) | 23.0 (21.0-26.0) |
| Gestational age at L&D admission in weeks, median (IQR) | 36.1 (34.6-38.9) | 33.1 (29.3-35.2) | | 34.3 (31.9-36.1) | 36.7 (35.3-39.4) |
| Multiple gestation, n (%) | 48 (5.1) | 1 (10.0) | | 28 (11.8) | 19 (2.7) |
| Pre-gestational diabetes, n (%) | 7 (0.7) | 0 (0) | | 5 (2.1) | 2 (0.3) |
| Gestational diabetes, n (%) | 16 (1.7) | 0 (0) | | 6 (2.5) | 10 (1.4) |
| Preeclampsia, n (%) | 29 (3.1) | 0 (0) | | 17 (7.2) | 12 (1.7) |
| Antenatal steroids within 14 days of admission, n (%) | 505 (53.8) | 7 (70.0) | | 173 (73.0) | 325 (47.0) |
| Antepartum antibiotics, n (%) | 737 (78.5) | 7 (70.0) | | 180 (75.9) | 550 (79.5) |
| PROM, n (%) | 362 (38.6) | 4 (40.0) | | 95 (40.1) | 263 (38.0) |
| ROM in hours, median (IQR) | 12.0 (5.0-22.0) | 16.5 (13.2-18.8) | | 14.0 (6.0-22.5) | 12.0 (5.0-22.0) |
| Meconium-stained amniotic fluid, n (%) | 288 (30.7) | 1 (10.0) | | 32 (13.5) | 255 (36.8) |
| Number of vaginal exams prior to delivery, median (IQR) | 1.0 (1.0-2.0) | 1.0 (1.0-2.0) | 1.0 (1.0-2.0) | | 1.0 (1.0-2.0) |
| Cesarean delivery, n (%) | 329 (35.0) | 1 (10.0) | 74 (31.2) | | 254 (36.7) |

Supplemental Table 1 notes. Characteristics of 939 women with liveborn neonates by disposition of neonate, neonatal death in delivery room, NICU admission, and routine newborn care. Abbreviations: DR – delivery room; IQR – interquartile range; L&D – Labor and Delivery; NICU – neonatal intensive care unit; PROM – premature rupture of membranes; ROM – rupture of membranes.

**Supplementary Table 2. Gram-negative blood culture species identification by cohort, bacteremia onset, and antimicrobial susceptibility testing**

| Bacterial species | Total  (n=57) | Cohort A  (n=21) | Cohort B  (n=36) | Early onset  (n=10) | Late onset  (n=47) | Non-susceptible to 3^rd^/4^th^ generation cephalosporins or piperacillin-tazobactam | Non-susceptible to carbapenems |
| --- | --- | --- | --- | --- | --- | --- | --- |
| *Acinetobacter* spp., n (%) | 5 (9) | 2 (9.5) | 3 (8.3) | 1 (10.0) | 4 (8.5) | 3 (60) | 2 (40) |
| *Burkholderia cepacia*, n (%) | 3 (5) | 3 (14.3) | 0 | 1 (10.0) | 2 (4.3) | 2 (67) | 0 (0) |
| *Citrobacter* spp., n (%) | 5 (9) | 2 (9.5) | 3 (8.3) | 0 | 5 (10.6) | 3 (60) | 1 (20) |
| *Enterobacter* spp., n (%) | 5 (9) | 1 (4.8) | 4 (11.1) | 0 | 5 (10.6) | 3 (60) | 1 (20) |
| *Escherichia coli*, n (%) | 5 (9) | 0 | 5 (13.9) | 1 (10.0) | 4 (8.5) | 5 (100) | 1 (20) |
| *Klebsiella* spp., n (%) | 25 (44) | 8 (38.1) | 17 (47.2) | 5 (50.0) | 20 (42.6) | 22 (88) | 15 (60) |
| *Pseudomonas* spp., n (%) | 9 (16) | 5 (23.8) | 4 (11.1) | 2 (20.0) | 7 (14.9) | 5 (56)* | 2 (22) |

Supplementary Table 2 notes. Cohort A includes neonates born to mothers prospectively enrolled prior to delivery; Cohort B includes neonates admitted to the NICU during the study period but not enrolled in the maternal colonization study and observed for BSI as part of a parent cohort study. Early onset infections include 5 from Cohort A and 5 from Cohort B. Late onset infections include 16 from Cohort A and 31 from Cohort B. Abbreviations: *Spp.* – species.

**Supplementary Table 3. Plasmids detected by species among Enterobacterales isolates**

|  | Specimens, n with plasmid group | | |
| --- | --- | --- | --- |
| Plasmid Group | *Enterobacter hormaechei*, n = 2 | *Escherichia coli*,  n = 4 | *Klebsiella pneumoniae,* n = 19 |
| Col_BS512 | 0 | 1 | 0 |
| Col_pHAD28 | 2 | 1 | 4 |
| Col156 | 0 | 1 | 0 |
| Col440I | 0 | 1 | 14 |
| Col440II | 1 | 0 | 3 |
| Col8282 | 0 | 0 | 0 |
| ColKP3 | 0 | 0 | 6 |
| ColpVC | 0 | 0 | 12 |
| ColRNAI | 0 | 2 | 0 |
| FIA_pBK30683 | 0 | 0 | 5 |
| IncB | 0 | 0 | 0 |
| IncC | 0 | 0 | 3 |
| IncFIA | 0 | 2 | 0 |
| IncFIB_AP001918 | 0 | 3 | 0 |
| IncFIB_H89_PhagePlasmid | 0 | 0 | 0 |
| IncFIB_K | 0 | 0 | 18 |
| IncFIB_K_pCAV1099_114 | 0 | 0 | 0 |
| IncFIB_pKPHS1 | 0 | 0 | 1 |
| IncFIB_pNDM_Mar | 0 | 0 | 3 |
| IncFIB_pQil | 0 | 0 | 16 |
| IncFII | 0 | 1 | 2 |
| IncFII_K | 0 | 0 | 13 |
| IncFII_pAMA1167_NDM_5 | 0 | 0 | 0 |
| IncFII_pKP91 | 0 | 0 | 14 |
| IncFII_pKPX1 | 1 | 0 | 0 |
| IncFII_pRSB107 | 0 | 1 | 0 |
| IncHI1B_pNDM_MAR | 0 | 0 | 3 |
| IncHI2 | 0 | 0 | 3 |
| IncHI2A | 0 | 0 | 3 |
| IncI_Gamma | 0 | 1 | 0 |
| IncI1_I_Alpha | 0 | 0 | 0 |
| IncM2 | 0 | 0 | 0 |
| IncQ1 | 0 | 1 | 0 |
| IncR | 0 | 0 | 5 |
| IncU | 0 | 0 | 3 |
| IncX1 | 0 | 1 | 0 |
| IncX4 | 0 | 0 | 0 |
| IncY | 0 | 1 | 0 |
| mcr_9 | 0 | 0 | 3 |

*Enterobacter hormaechei* isolates are all from Cohort A including one identified as *Citrobacter* species by conventional identification. *Escherichia coli* isolates are all from Cohort B. *Klebsiella pneumoniae* are from both cohorts (Cohort A, n = 6; Cohort B, n = 13).

**Supplementary Figure 3. Strain relatedness, bacteremia onset, and select antimicrobial resistance (AMR) genes for neonates with *Escherichia coli* (A), *Burkholderia cenocepacia* (B), and *Acinetobacter baumannii* (C) bloodstream infections.** Strain relatedness is expressed on the left as a rooted maximum likelihood phylogenetic tree for isolate collections with ≥4 specimens; isolate collections with <4 specimens are shown with a hierarchical cluster tree. Following is the sequence type (ST) assigned by multilocus sequence typing (MLST). Next to the right, pairwise single-nucleotide polymorphisms (SNPs) between each pair of isolates is presented as a rotated distance matrix with the diagonal aligned with the corresponding isolate from the phylogenetic tree. Corresponding period of hospitalization and bacteremia onset are displayed on the right followed by select AMR genes. *Escherichia coli* isolates are all from Cohort B. *Burkholderia cenocepacia* isolates are from Cohort A (n = 3) and Cohort B (n=1, identified as *Pseudomonas aeruginosa* by conventional identification). *Acinetobacter baumannii* isolates are from Cohort A (n=2) and Cohort B (n=1).

**A. *Escherichia coli***
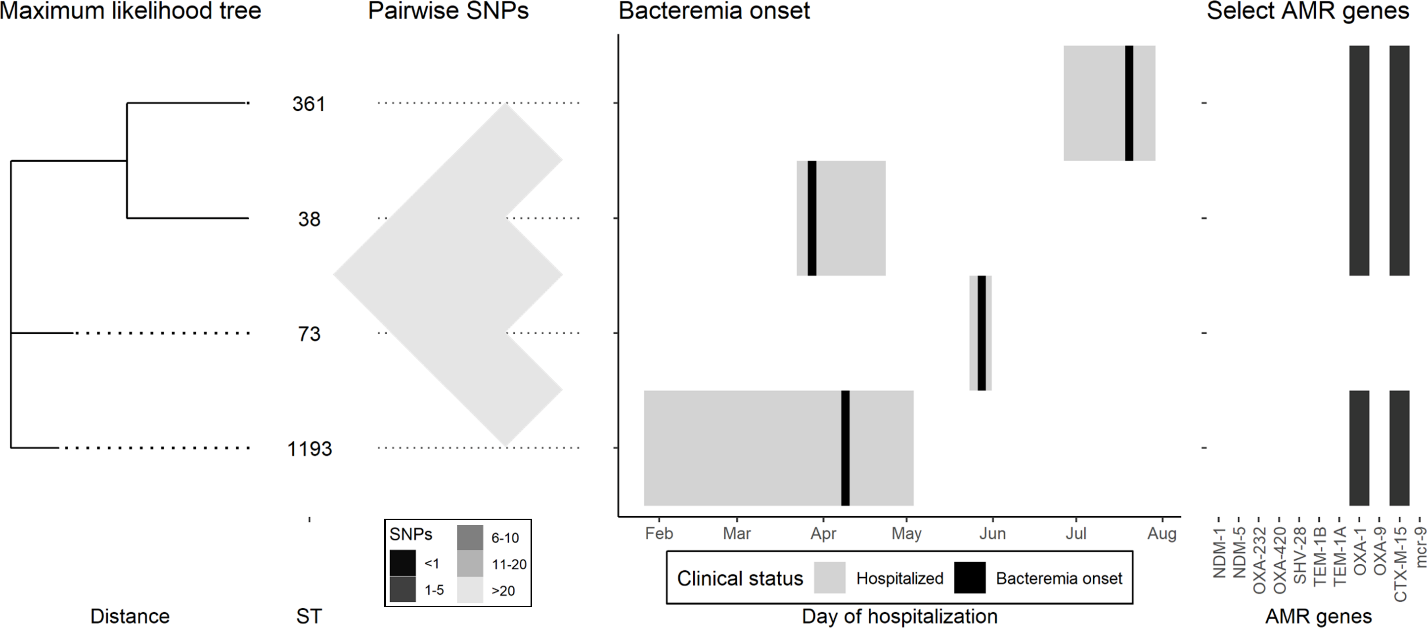


1. ***Burkholderia cenocepacia***


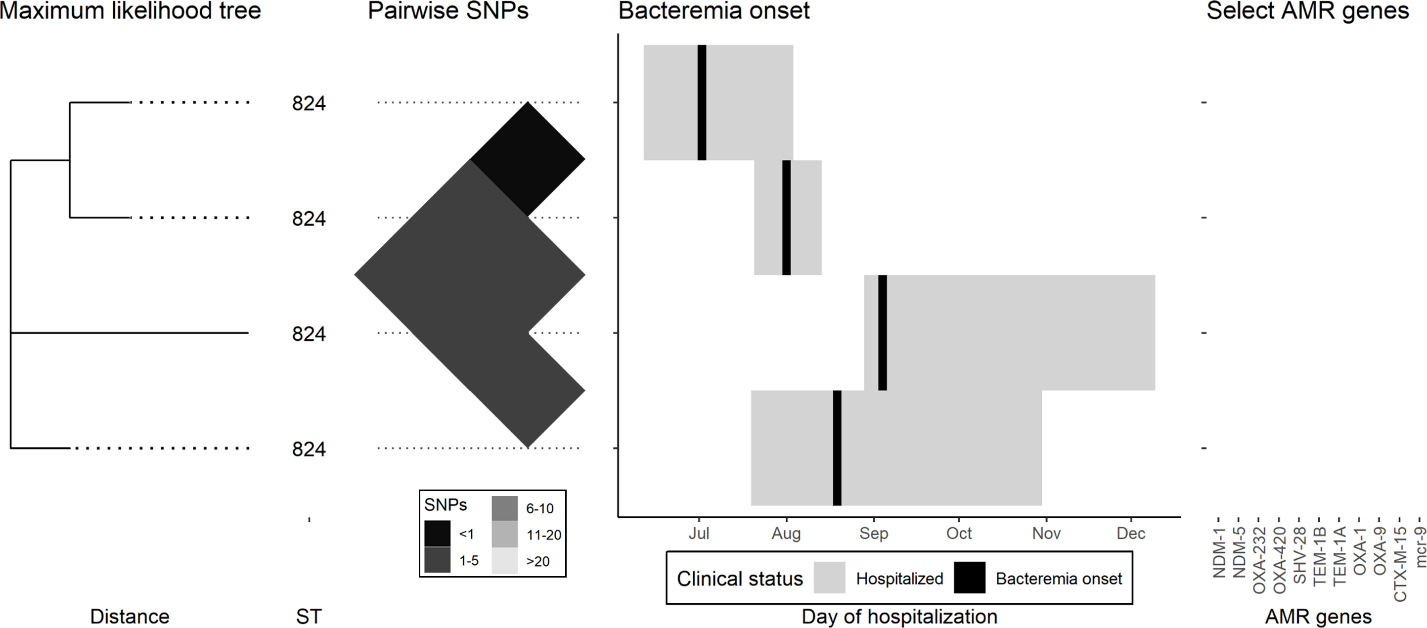


1. ***Acinetobacter baumannii***


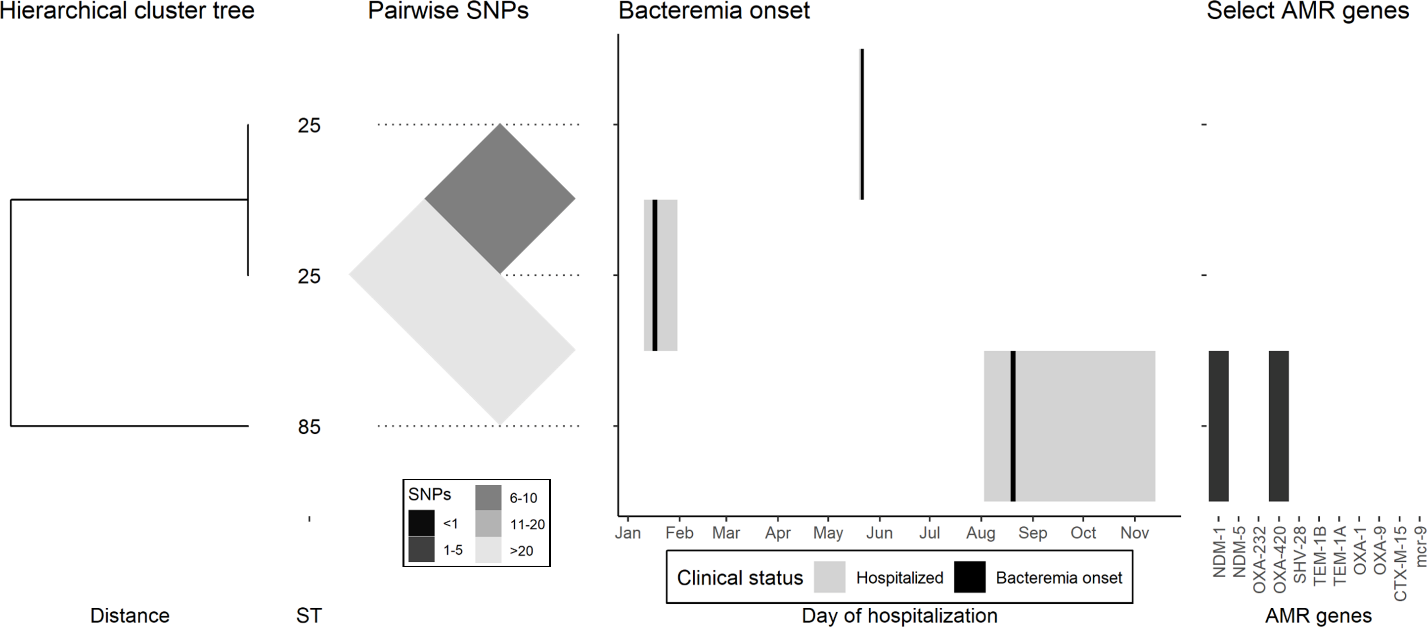

Supplement: ciad282_Supplementary_Data [file ciad282_supplementary_data.docx]
